# Altered Hippocampal Epigenetic Regulation Underlying Reduced Cognitive Development in Response to Early Life Environmental Insults

**DOI:** 10.3390/genes11020162

**Published:** 2020-02-04

**Authors:** Kyle M. Schachtschneider, Michael E. Welge, Loretta S. Auvil, Sulalita Chaki, Laurie A. Rund, Ole Madsen, Monica R.P. Elmore, Rodney W. Johnson, Martien A.M. Groenen, Lawrence B. Schook

**Affiliations:** 1 Department of Radiology, University of Illinois at Chicago, Chicago, IL 60607, USA; kschach2@uic.edu; 2 Department of Biochemistry and Molecular Genetics, University of Illinois at Chicago, Chicago, IL 60607, USA; 3 National Center for Supercomputing Applications, University of Illinois at Urbana-Champaign, Urbana, IL 61820, USA; mwelge@illinois.edu (M.E.W.); lauvil@illinois.edu (L.S.A.); 4 Department of Animal Sciences, University of Illinois at Urbana-Champaign, Urbana, IL 616280, USA; schaki@illinois.edu (S.C.); larund@illinois.edu (L.A.R.); elmorem@uci.edu (M.R.P.E.); rwjohn@illinois.edu (R.W.J.); 5 Animal Breeding and Genomics, Wageningen University, 6708 Wageningen, The Netherlands; ole.madsen@wur.nl (O.M.); martien.groenen@wur.nl (M.A.M.G.)

**Keywords:** porcine biomedical models, hippocampus, cognitive development, DNA methylation, RNA-seq, machine learning

## Abstract

The hippocampus is involved in learning and memory and undergoes significant growth and maturation during the neonatal period. Environmental insults during this developmental timeframe can have lasting effects on brain structure and function. This study assessed hippocampal DNA methylation and gene transcription from two independent studies reporting reduced cognitive development stemming from early life environmental insults (iron deficiency and porcine reproductive and respiratory syndrome virus (PRRSv) infection) using porcine biomedical models. In total, 420 differentially expressed genes (DEGs) were identified between the reduced cognition and control groups, including genes involved in neurodevelopment and function. Gene ontology (GO) terms enriched for DEGs were associated with immune responses, angiogenesis, and cellular development. In addition, 116 differentially methylated regions (DMRs) were identified, which overlapped 125 genes. While no GO terms were enriched for genes overlapping DMRs, many of these genes are known to be involved in neurodevelopment and function, angiogenesis, and immunity. The observed altered methylation and expression of genes involved in neurological function suggest reduced cognition in response to early life environmental insults is due to altered cholinergic signaling and calcium regulation. Finally, two DMRs overlapped with two DEGs, *VWF* and *LRRC32*, which are associated with blood brain barrier permeability and regulatory T-cell activation, respectively. These results support the role of altered hippocampal DNA methylation and gene expression in early life environmentally-induced reductions in cognitive development across independent studies.

## 1. Introduction

The hippocampus is a brain region important for learning, memory, and emotional responses [1]. In addition to brain development that occurs during the prenatal period, significant brain growth and maturation including synaptogenesis, dendritic growth, and glial cell proliferation also occurs during the first few years of life [1,2]. Environmental insults during this period can have lasting effects on brain structure and function [3,4,5,6], with the hippocampus displaying high susceptibility to the effects of early life environmental insults [7]. Previous studies have identified altered gene expression in the hippocampus of individuals with altered functions, such as reduced learning and memory or behavioral changes [8,9,10,11,12]. However, the cellular mechanisms responsible for altered gene expression in response to early life environmental insults are largely unknown.

The field of epigenetics focuses on gene expression and phenotypic changes that develop without changes to an individual’s DNA sequence [13]. One of the most well understood epigenetic marks is DNA methylation, which occurs predominantly at CpG sites—defined as a cytosine nucleotide followed by a guanine nucleotide—throughout the genome. DNA methylation levels play an important role in gene expression and are affected by environmental exposures during development [13]. Due to the known relationship between environmental exposures, DNA methylation, and gene regulation, it is expected that aberrant gene expression resulting from altered DNA methylation levels can help explain phenotypic changes induced by environmental exposures. However, extracting multi-omics signatures from high-dimensional heterogeneous data presents unique analytical challenges because these data belong to the p>>n class of problems where the number of features is orders of magnitude larger than the number of samples in the study [14,15]. Likewise, the common univariate approaches for ranking feature relevance fail to capture complex multivariate relationships and apply feature-ranking criteria unrelated to the model accuracy. These approaches can be unstable and result in high false discovery rates and unreproducible predictive models [16,17,18].

In contrast, more promising approaches from the domain of machine learning have been proposed for prediction, feature selection, and feature ranking in fields related to computational biomedicine [19]. A particularly well-suited machine learning method to address signature extraction is random forest [20], a collaborative ensemble learning method based on decision trees that has been successfully applied in genomics [21], gene expression [22], methylation [23], proteomics [24], and metabolomics studies [25]. Random forest provides variable importance measures, which can be used to select and rank features based on their predictive importance. However, it is difficult to distinguish relevant from irrelevant variables based on the random forest variable importance measure alone [26,27,28]. In recent studies of popular alternative approaches where the goal is to find *all relevant features*, Boruta has been the best performing approach [26,27,28,29]. Boruta is a random forest approach that identifies the most important features with high feature selection stability [30,31]. Boruta has been used in over 100 studies to date, including omics datasets comprising gene expression, DNA methylation [32], and microbiome data [33], as well as previous studies by the research team [34,35,36,37,38].

Recently, the neonatal pig has emerged as a model for studying how early life environmental insults affect brain development and behavior due to similarities between porcine and human neurodevelopment [39,40]. For example, the aforementioned neonatal brain growth spurt is observed in both humans and pigs. In addition, piglets and human infants display similarities in gyral patterning and distribution of gray and white matter [41,42], and behavioral testing can be performed to assess cognitive development in pigs as early as 1–2 weeks of age [43,44]. Finally, porcine DNA methylation distribution and functional patterns are similar to those observed in humans [45,46], making them ideal for studying not only the effects of early life environmental insults on spatial learning and memory, but the potential epigenetic mechanisms underlying the altered phenotype.

This study utilized Boruta machine learning approaches to identify altered hippocampal DNA methylation and gene expression patterns from two previously published studies reporting reduced hippocampal-based spatial learning and memory in response to two early life environmental insults; neonatal peripheral viral infection with the porcine reproductive and respiratory syndrome virus (PRRSv) [39,47] and neonatal iron deficiency [40,48]. These studies were chosen due to the similar reduced cognitive development phenotypes observed despite the use of different environmental insults (nutrient deficiency and viral infection), with the goal of identifying the molecular mechanisms through which two distinct environmental insults result in the same reduced cognition phenotype.

## 2. Materials and Methods

### 2.1. Ethics Statement

Tissue collection was conducted at in accordance with national and international guidelines and approved by The University of Illinois Institutional Animal Care and Use Committee (IACUC protocol number 10189 and 10163).

### 2.2. Sample Collection

Hippocampus samples were collected from 4 week old female Landrace, Large White, Duroc, and Pietran mixed piglets from a previously published study [39]. Briefly, PRRSv infected piglets were inoculated intranasally with live PRRSv at 7 days of age, and PRRSv and control piglets were euthanized at 4 weeks of age [39]. The samples were rinsed with phosphate buffered saline (PBS) and stored at −80 °C until processing.

### 2.3. DNA Isolation

DNA was isolated from frozen PRRSv infected (*n* = 4) and control (*n* = 5) hippocampus tissue samples using the AllPrep DNA/RNA Mini Kit (Qiagen, Valencia, CA, USA) and assessed for quality as previously described [48]. The RNA and DNA used to produce RNA-seq and reduced representation bisulfite sequencing (RRBS) datasets utilized in this study was simultaneously extracted from each sample using the AllPrep DNA/RNA Mini Kit (Qiagen, Valencia, CA, USA) in order to allow for assessment of gene expression and DNA methylation levels of the same cellular population.

### 2.4. RRBS and Targeted Control Library Preparation

High-quality DNA (2 μg) was provided to the Carver High-Throughput DNA Sequencing and Genotyping Unit (HTS lab, University of Illinois, Urbana, IL, USA) for generation of RRBS and targeted control libraries as previously described [45].

### 2.5. Illumina Sequencing

Single end 100 bp reads were produced on an Illumina HiSeq2500 for the RRBS and targeted control libraries by the HTS lab. The data sets supporting the results of this article are available in the European Nucleotide Archive under accession number PRJEB11625 (www.ebi.ac.uk/ena/data/view/PRJEB11625).

### 2.6. RRBS Data Analysis

On average 45.9 million reads were produced for each library (range of 29.4 to 68.5 million). RRBS datasets produced from frozen hippocampus samples of 4 week old iron deficient (*n* = 3) and control piglets (*n* = 4) were downloaded from the European Nucleotide Archive (ENA) database (www.ebi.ac.uk/ena) under accession number PRJEB12278 [48]. Raw reads were trimmed for adapters, quality, length, and experimentally introduced cytosines as previously described [48]. Trimmed reads were aligned to the swine reference genome (Sscrofa10.2 [49]) and methylation levels were extracted using BS-seeker2 v.2.0.5 [50] as previously described [48]. The ratio of unmethylated to total reads for covered cytosines on the mitochondrial genome was calculated to determine the bisulfite conversion rate (98.6%). In order to reduce potential sequencing depth biases, downstream analyses were limited to sites covered by a minimum of 10 reads across all samples (high confidence sites). Due to the low coverage of the RRBS dataset for sample 40 in the PRRSv infection study, this sample was removed before performing DNA methylation analysis. High confidence CpG sites were utilized to define CpG methylation regions using the DMRfinder V0.3 combine_CpG_sites.py script with default parameters [51]. Identified DNA methylation regions were then filtered to select regions located within gene regions, including up to 10 kb upstream of gene transcription start sites (TSS). The distribution of methylation region sizes and distance from TSS are depicted in Figure S1. For each region, percent methylation was used for comparative analyses.

### 2.7. Targeted Control Data Analysis

Targeted control datasets were used for SNP detection as previously described [45]. On average, 14.6 million reads were produced for each library (range of 11.1 to 17.9 million). In addition, previously produced targeted control datasets were downloaded from the ENA database (www.ebi.ac.uk/ena) under accession number PRJEB12278 for the iron deficiency study samples [48]. Trimming, alignment, and variant calling was performed as previously described [45], resulting in an average of 1.33% of the genome covered at a depth of 7.13. On average, 928,703 (63.76%) CpG sites/individual were covered by both the RRBS and targeted control datasets, resulting in the detection of 31,346 (average of 11,341/individual) SNPs. CpG sites containing SNPs were removed prior to performance of downstream analyses.

### 2.8. RNA-Seq Analysis

Previously produced RNA-seq datasets were downloaded from the ENA database (www.ebi.ac.uk/ena) under accession number PRJEB12278 for the iron deficiency samples and PRJEB11625 for the PRRSv infection samples [47,48]. Sequencing reads were processed, aligned to Sscrofa10.2 [49], Ensembl release 81, and gene expression quantified as previously described [47,48]. Gene expression was normalized across samples using Cuffnorm v.2.2.1 with the –library-type fr-firststrand and –library-norm-method geometric options [52]. FPKM values were used for comparative analyses.

### 2.9. Machine Learning

We leveraged the BorutaPy codebase (https://github.com/scikit-learn-contrib/boruta_py), which uses the random forest algorithm from SciKit Learn (https://scikit-learn.org/) to identify all relevant features. The Boruta algorithm produces three sets of features: confirmed features that are relevant, rejected features that are irrelevant, and tentative features that cannot be confirmed or rejected. We used the National Center for Supercomputing Applications (NCSA) Visual Intelligence for Biology (VI-Bio) analytical pipeline to call the BorutaPy algorithm. The Boruta package allows random forest tree parameters to be manipulated. Defaults parameters were used to independently analyze the DNA methylation and gene expression datasets, with the maximum tree depth set to 4, number of estimators set to 10,000, percentile to pick our threshold for comparison between shadow and real features set to 95, and maximum iterations set to 50. In total, 30,468 DNA methylation regions were tested, with Boruta identifying 608 confirmed differentially methylated regions (DMRs) and another 335 tentative DMRs between the reduced cognition (PRRSv infected and iron deficient pigs) and control pigs. Confirmed and tentative DMRs were combined and filtered to exclude regions with a difference in methylation level of less than 10% between groups, resulting in a total of 116 DMRs. In total, 38,219 genes were tested, with Boruta identifying 863 confirmed differentially expressed genes (DEGs) and another 880 tentative DEGs. Confirmed and tentative DEGs were combined and filtered to exclude genes with a log2 fold change between 1 and −1 between groups, resulting in a total of 420 DEGs.

### 2.10. Statistics

Correlations were investigated using the Pearson correlation coefficient, and Bray-Curtis dissimilarities were used to perform analysis of similarities (ANOSIM) analyses. R v.3.1.2 [53] was used to perform all statistical tests.

### 2.11. GO Term Enrichment Analysis

Gene ontology (GO) term enrichments were determined using the Gene Ontology Consortium enrichment analysis tool [54,55,56]. The biological process domain was used to perform the GO term analysis, and *p*-values were corrected for multiple testing using the false discovery rate (FDR). GO terms with a *q*-value < 0.05 were considered enriched.

## 3. Results

### 3.1. Early Life Environmental Insults Result in Altered Epigenetic and Transcriptional Patterns

**Figure 1:**
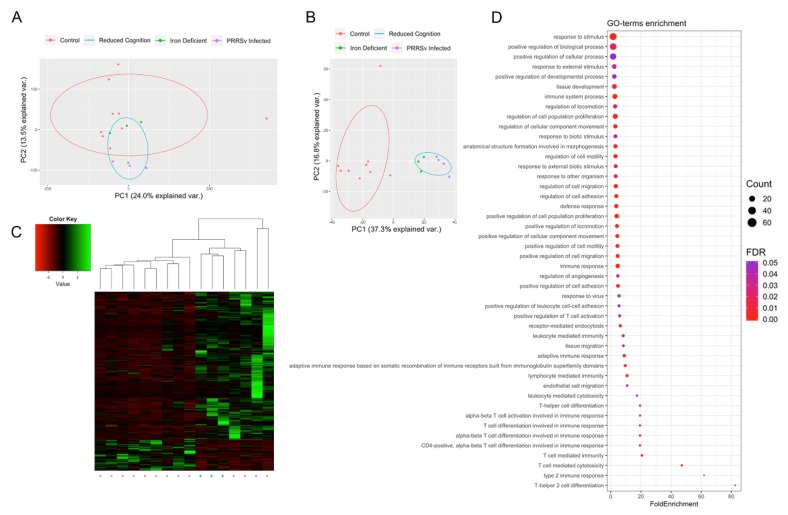
Alteration of Hippocampal Gene Expression Patterns. Principle component analysis (PCA) based on the expression level of (**A**) all tested genes and (**B**) DEGs. (**C**) Heatmap of the expression levels of the 420 DEGs, represented as z-scores. (**D**) Identified GO terms enriched for DEGs.

Altered gene expression between the reduced cognition (PRRSv infected and iron deficient pigs) and control groups was assessed, resulting in the identification of 420 DEGs, with 72 down- and 348 upregulated in the reduced cognition compared to control group. While samples did not cluster by group when comparing expression levels of the 38,219 tested genes (ANOSIM R = 0.1189, *p*-value = 0.075; Figure 1A), samples did cluster by group when comparing expression levels of the 420 DEGs (ANOSIM R = 0.6981, *p*-value = 0.001; Figure 1B,C). GO term enrichment analysis resulted in identification of 46 GO terms enriched for DEGs (Figure 1D). Enriched GO terms were associated with immune responses, angiogenesis, cellular development, migration, and proliferation.

**Figure 2:**
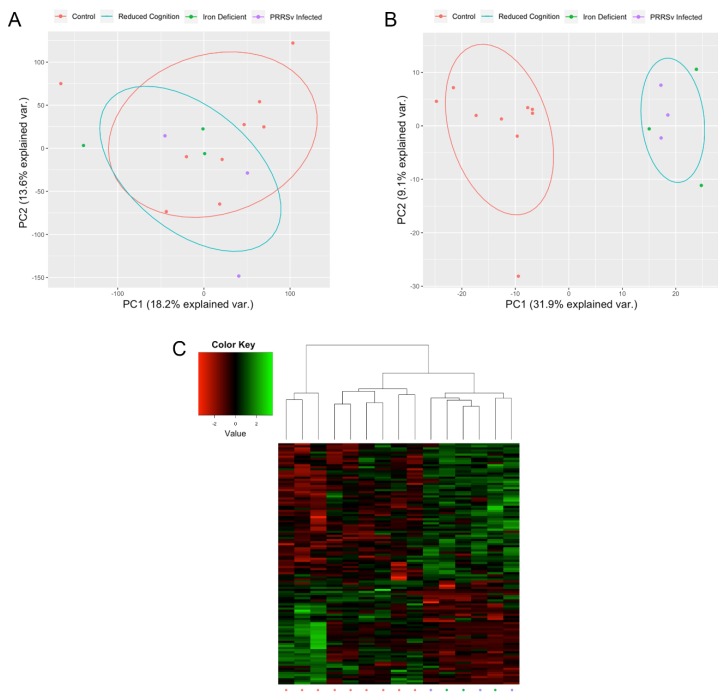
Altered Hippocampal DNA Methylation Patterns. PCA based on the methylation level of (**A**) all tested regions and (**B**) DMRs. (**C**) Heatmap of the methylation levels of the 116 DMRs, represented as z-scores.

In addition to altered gene expression, 116 genomic regions with significant DNA methylation differences between the reduced cognition and control groups were identified, with 50 hypo- and 66 hypermethylated in the reduced cognition compared to control group. While samples did not cluster by group when comparing methylation levels of the 30,468 tested regions (ANOSIM R = −0.06899, *p*-value = 0.731), samples did cluster by group based on methylation levels of the 116 DMRs (ANOSIM R = 0.7741, *p*-value = 0.001; Figure 2). The 116 DMRs overlapped with 125 genes. GO term enrichment analysis did not identify any GO terms enriched for genes overlapping DMRs. Of the 125 genes overlapping DMRs, two genes (*VWF* and *LRRC32*) were identified as DEGs, each of which overlapped with a single intragenic DMR.

### 3.2. Activation of Immune Responses

**Figure 3:**
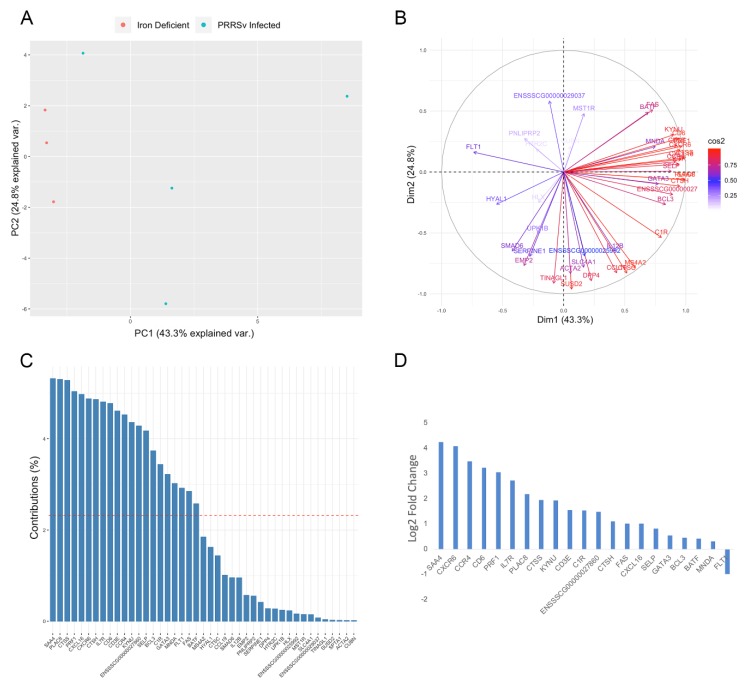
Contribution of immune related DEGs to reduced cognition group variation. (**A**) PCA based on the expression level of 43 immune related DEGs. (**B**) Breakdown of variables contributing to each principle component. (**C**) Percent contribution of each gene to principle component 1 (PC1). The red dotted line signifies the expected contribution of each gene, and any genes contributing at a higher rate were considered significant contributors. (**D**) Sixteen DEGs contributing to PC1 displayed a log2 fold change differences >1 or <−1. Log2 fold changes represent expression levels in the PRRSv infected compared to iron deficient group.

A number of identified DEGs, as well as the majority of GO terms enriched for DEGs were associated with activation of immune responses. Immune related GO terms enriched for DEGs included T-helper 2 cell differentiation, T cell mediated cytotoxicity, positive regulation of T cell activation, response to virus, and defense response (Figure 1D). This result was expected in the case of pigs exposed to early life viral infection; however, pigs exposed to early life iron deficiency were not expected to display significant immune activation. Therefore, we compared samples based on the expression of DEGs associated with enriched immune response GO terms. Samples did not cluster separately when assessing the expression level of the 43 immune-related DEGs (ANOSIM R = 0.1296, *p*-value = 0.29; Figure 3A). Although significant differences between the iron deficient and PRRSv infected group were not identified, the two groups appear to be most clearly separated by PC1 (Figure 3A). Therefore, we identified genes contributing the most to PC1 (Figure 3B,C). A total of 21 genes were found to contribute to PC1 at a rate higher than expected by chance (Figure 3C). Of these, 15 displayed a log2 fold change >1 in the PRRSv infected compared to iron deficient group, with only one gene (*FLT1*) expressed at a higher level in the iron deficient group (Figure 3D). *FLT1* is a vascular endothelial growth factor receptor that regulates angiogenesis and VEGF-induced monocyte migration [57], and therefore, increased expression in the iron deficient pigs could be due to its role in angiogenesis as opposed to immunity. The five DEGs with the highest log2 fold change increase in the PRRSv infected compared to iron deficient group belong to the defense response GO term, defined by reactions triggered in response to the presence of a foreign body or injury. We therefore concluded that the enrichment of GO terms associated with immune responses is likely due to the response to PRRSv in the PRRSv infected group.

**Figure 4:**
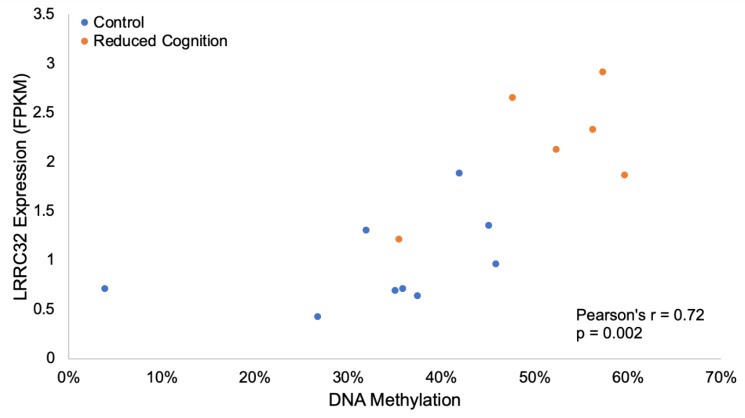
Differential methylation and expression of *LRRC32.* Hypermethylation of a 60 bp region in the 2nd intron of *LRRC32* correlated with increased expression in the reduced cognition group (Pearson’s R = 0.72, *p*-value = 0.002).

One gene (*LRRC32*) related to immune responses was both differentially methylated and expressed in this study. *LRRC32* is a key regulator of TGF-β and is expressed by activated T regulatory (T_reg_) cells [58]. Hypermethylation of a 60 bp region in the 2nd intron of *LRRC32* correlated with increased expression in the reduced cognition group (Pearson’s R = 0.72, *p*-value = 0.002; Figure 4). The increased *LRRC32* methylation and expression in the reduced cognition group suggests higher levels of T_reg_ cells in the hippocampus of pigs suffering from reduced cognitive development. This is in contrast to previous reports demonstrating neuroprotective effects of T_regs_ in mouse models of Alzheimer’s disease, including reduced spatial learning observed following depletion of T_regs_ [59]. Neuroimmune factors are also thought to play important roles in the etiology of psychiatric disorders such as autism spectrum disorders and schizophrenia [47]. Together, these results suggest aberrant immune system activation represents one of the mechanisms underlying the reduced cognition phenotype.

### 3.3. Increased Angiogenesis and Blood Brain Barrier Permeability

A number of GO terms related to angiogenesis were enriched for DEGs, including regulation of angiogenesis, anatomical structure formation involved in morphogenesis, and endothelial cell migration. Consistent with our previous publication demonstrating increased expression of proangiogenic factors in the iron deficient group [48], increased expression of the proangiogenic factors *ENPP2*, *FLT1*, and *ADM* was observed in the reduced cognition group. Other positive regulators of angiogenesis with increased expression in the reduced cognition group included *EMP2*, *CTSH*, *HYAL1*, *SRPX2*, *ADM*, *COL8A1*, and *PGF*. However, increased expression of negative regulators of angiogenesis was also observed in the reduced cognition group, including *MEOX2*, *COL18A1*, *ADAMTS1*, and *MMRN2*. Due to the increased expression of both positive and negative regulators of angiogenesis, it is unclear whether these changes indicate increased or decreased angiogenesis in response to early life environmental insults. However, the altered expression, in addition to the identification of DMRs overlapping genes associated with angiogenesis, including *VAV2*, *ILK*, *PITPNM3*, and *AMOTL1*, suggests alteration in the natural neonatal angiogenic process in the hippocampus of the reduced cognition group.

**Figure 5:**
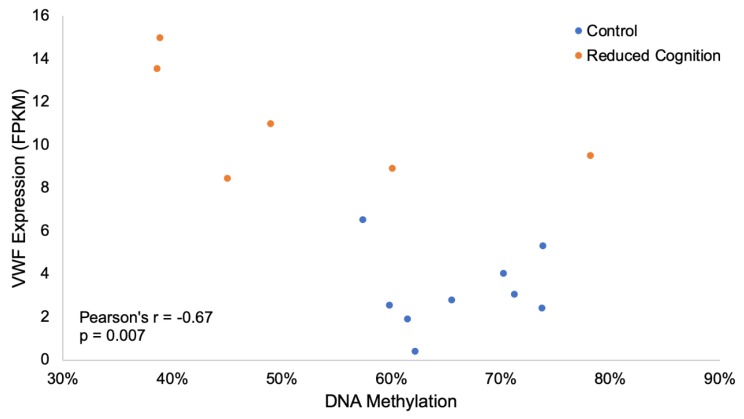
Differential methylation and expression of *VWF.* Hypomethylation of a 28 bp region in the 11^th^ intron of *VWF* correlated with increased expression in the reduced cognition group (Pearson’s R = −0.67, *p*-value = 0.007).

In addition to altered angiogenic regulation, DEGs involved in blood brain barrier (BBB) formation and permeability were also identified. *AHNAK* plays a role in BBB formation [60], and was overexpressed in the reduced cognition group (log2 fold change 1.03). *VWF* and *FLT1* expression are associated with BBB hyperpermeability [61,62], both of which displayed increased expression in the reduced cognition group (log2 fold change 1.82 and 1.01, respectively). In addition, hypomethylation of a 28 bp region (−14.54%) in the 11th intron of *VWF* correlated with increased expression in the reduced cognition group (Pearson’s R = −0.67, *p*-value = 0.007; Figure 5). This result is consistent with our previous study demonstrating hypomethylation of a CpG site within the same 28 bp region associated with increased *VWF* expression in the iron deficient group [48]. Together, the altered regulation and expression of *VWF*, in addition to increased *FLT1* expression, suggests increased BBB permeability in the reduced cognition groups in response to early life environmental insults.

### 3.4. Altered Neurodevelopment and Function

Although no GO terms related to neurodevelopment and function were enriched for DEGs, differential expression or methylation of genes associated with neurodevelopmental disorders or learning disabilities was observed in this study. *FOXP2* is a transcription factor expressed in neuronal tissue and is involved in synapse formation by regulating *SRPX2* levels [63]. Together, these genes play a role in proper development of speech and language [63,64]. *FOXP2* is also important for modulating plasticity of neural circuits [65]. Both *FOXP2* and *SRPX2* displayed increased expression in the reduced cognition compared to control hippocampus samples (log2 fold change 1.02 and 1.62, respectively). Increased expression of the serotonin receptor *HTR2C* was also observed in the reduced cognition group (log2 fold change 1.18), consistent with our previous publication demonstrating increased expression in the iron deficient group [48]. Serotonin is a neurotransmitter involved in many behavioral and functional processes including pain, emotion, and learning and memory [66]. Increased *HTR2C* expression has also been identified in individuals suffering from depression and Huntington’s disease [67,68]. Increased expression of *PMCH* and *DRD1*, both of which are involved in regulating behavioral responses [69,70,71], was observed in the reduced cognition group (log2 fold change 1.23 and 1.55, respectively). Finally, *NTS* has been implicated in a number of neurological disorders including schizophrenia, drug abuse, and Parkinson’s disease [72], and was overexpressed in the reduced cognition group (log2 fold change 2.67).

DMRs overlapping genes involved in neurological disorders were also identified, including hypomethylation of *SETBP1* (−28.23%). Mutations and expression changes in this gene are associated with speech impairments and mental retardation [73,74]. In addition, differential methylation of two genes associated with Alzheimer’s disease (*CHD5* and *DHCR24*) was also observed in this study. Hypomethylation of *CHD5* (−11.76%), which regulates genes implicated in aging and Alzheimer’s disease [75], and hypermethylation of *DHCR24* (10.39%), which has neuroprotective characteristics and is downregulated in neurons of Alzheimer’s disease patients [76] was observed in the reduced cognition group. Hypermethylation of *AADC*, which is crucial for brain development, was also observed in the reduced cognition group (10.84%). *AADC* deficiencies cause pediatric neuro-metabolic disease, which is associated with severe developmental delay [77]. *PHACTR1* mutations cause functional defects in neurons related to West syndrome and other intellectual disabilities [78], and was hypomethylated in the reduced cognition group (−19.15%). Differential methylation of genes associated with neuropsychiatric disorders was also observed, including hypomethylation of *CSMD2* (−13.75%), a synaptic transmembrane protein involved in development and maintenance of synapses [79], and hypermethylation of *ABLIM3* (18.49%), which is involved in axon guidance and has been associated with schizophrenia [80,81]. Finally, hypermethylation of the methylated DNA binding protein *MECP2* (17.20%) was observed in the reduced cognition group. *MECP2* acts as both a transcriptional activator and repressor and is associated with a number of neuropsychiatric disorders including Rett syndrome [82].

#### 3.4.1. Altered Cholinergic Signaling

Acetylcholine signaling underlies specific aspects of cognitive functions and behaviors, including learning and memory, with alterations in cholinergic signaling involved in the pathophysiology of multiple neuropsychiatric disorders [83]. A number of genes associated with cholinergic signaling were differentially expressed or methylated in this study. Nerve growth factor (*NGF; ENSSSCG00000021400*) is produced in the hippocampus and is essential for hippocampal plasticity and learning [84]. In this study, reduced *NGF* expression was observed in the reduced cognition group (log2 fold change −1.3). High *NGF* levels are maintained throughout life, with reduced *NGF* levels resulting in cholinergic and sympathetic neuronal atrophy [85,86,87,88]. This cholinergic neuronal atrophy is associated with spatial memory impairment disorders including Alzheimer’s Disease in humans and rodent models [89,90]. In addition, blockage of endogenous *NGF* significantly reduces hippocampal long-term potentiation and spatial memory in rats [84].

Previous studies have demonstrated ingrowth of sympathetic axons into the hippocampus in response to cholinergic neuronal atrophy, and this ingrowth rescues long-term depression induced by muscarinic M_1_ receptor activation [91]. *SEMA3G* displays chemorepulsive activities for sympathetic axons [92] and was upregulated in the reduced cognition group (log2 fold change 1.15), suggesting blockage of sympathetic axon ingrowth into the hippocampus triggered in response to cholinergic neuronal atrophy. We therefore hypothesize that reduced *NGF* expression in the reduced cognition group leads to cholinergic neuronal atrophy, and that overexpression of *SEMA3G* results in chemorepulsion of sympathetic axons.

**Figure 6:**
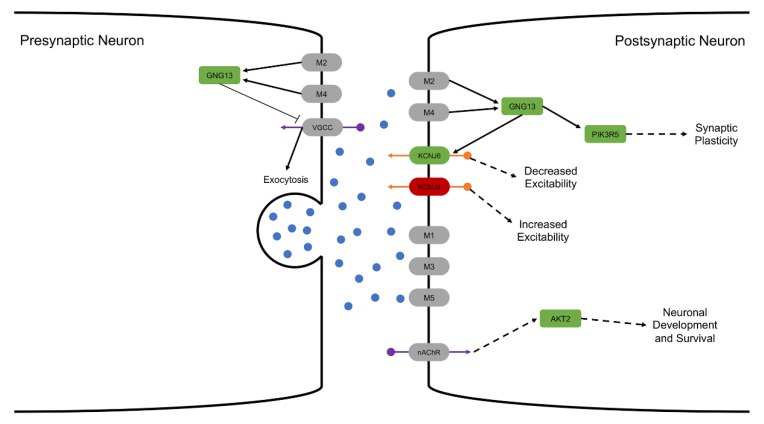
Altered methylation of genes in the cholinergic synapse pathway. Depiction of genes involved in cholinergic signaling. Green represents genes hypermethylated in the reduced cognition group, red represents genes hypomethylated in the reduced cognition group, and grey represents genes without differential methylation. Blue circles represent acetylcholine, orange circles represent potassium, and purple circles represent calcium.

While genes in the cholinergic synapse pathway were not differentially expressed, five genes in this pathway were differentially methylated (Figure 6). *GNG13* is a heterotrimeric G protein that functions as a signal transducer for 7-transmembrane-helix G protein-coupled receptors, including muscarinic acetylcholine receptors M2 and M4. *GNG13* was hypermethylated in the reduced cognition group (11.53%). *GNG13* activates *PIK3R5*, which was also hypermethylated in the reduced cognition group (10.23%). *PIK3R5* is one of the subunits that forms a complex with PI3K, which is an essential regulator of synaptic plasticity [93]. In addition, *GNG13* modulates the activity of G protein-coupled inwardly-rectifying potassium channels, including *KCNJ6* and *KCNJ4*, which were hyper- (11.09%) and hypomethylated (−14.77%) in the reduced cognition group, respectively. Finally, hypermethylation of *AKT2* was observed (10.02%). *AKT2* is a key modulator of the AKT-mTOR signaling pathway, which controls integration of newborn neurons during adult neurogenesis, including correct neuron positioning, dendritic development, and synapse formation in response to nicotinic acetylcholine receptor signaling. Together, these results suggest altered cholinergic signaling underlies the reduced cognitive development observed in response to early life environmental insults.

#### 3.4.2. Glutamate Transport and Calcium Regulation

Alterations related to glutamate transmembrane receptor regulation and signaling were observed, including hypermethylation of the glutamate transmembrane transporter *SLC1A7* in the reduced cognition group (11.6%). Glutamate induces an increase in the concentration of cytoplasmic calcium by directly activating α-amino-3-hydroxy-5-methyl-4-isoxazolepropionic acid (AMPA) receptor channels on neurons [94], and is a major excitatory transmitter in the brain [95]. AMPA receptors are glutamate-gated ion channels that mediate fast synaptic transmission that controls behavior and cognition [96]. *CACNG4* is a type I transmembrane AMPA receptor regulatory protein that was hypermethylated in the reduced cognition group (15.41%). *CACNG4* plays an important role in learning due to the importance of AMPA receptors for long term potentiation. *CACNG4* promotes AMPA receptor targeting to synapses and modulates their gating properties by slowing their rates of activation, deactivation, and desensitization [96].

**Figure 7:**
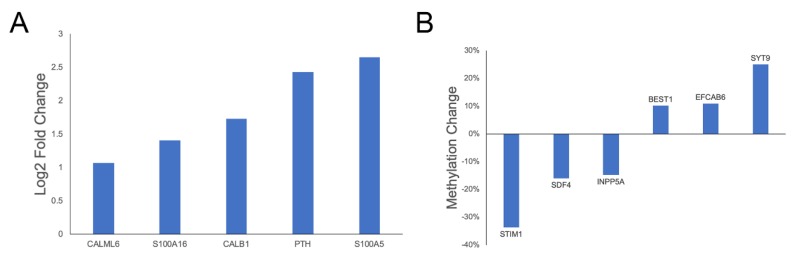
DEGs and genes overlapping DMRs related to calcium transport and binding. Log2 fold changes and percent methylation changes of (**A**) DEGs and (**B**) genes overlapping DMRs related to calcium transport and binding.

Transport of calcium across the plasma membrane by voltage-dependent and ligand-gated channels plays a critical role in fundamental neuronal activity including neurite outgrowth, synaptic transmission, and plasticity [94]. Both clinical and preclinical studies have implicated alterations in calcium regulation in the pathogenesis of chronic neurodegenerative disorders [94]. A number of genes involved in calcium transport and binding were found to be differentially expressed or methylated in this study (Figure 7), including increased expression of two S100 calcium binding genes, which are particularly interesting due to the close association between S100 genes and neurological disorders such as depression and Alzheimer’s disease [97]. Increased *CALB1* expression was also observed in the reduced cognition group (log2 fold change 1.73). *CALB1* buffers entry of calcium upon stimulation of glutamate receptors, and both increased and decreased hippocampal expression have been associated with reduced long-term potentiation and impaired spatial memory [98,99,100]. *CALB1* expression also affects memory performance in aged mice, most likely due to its role in the maintenance of neuronal calcium homeostasis, a critical factor in synaptic transmission and plasticity [94,98].

In addition to altered expression, hypomethylation of *STIM1*, which mediates calcium influx following depletion of intracellular calcium stores [101], was observed in the reduced cognition group (−33.6%). *BEST1* regulates voltage-gated calcium-ion channels expressed in hippocampal neurons and was hypermethylated in the reduced cognition group (10.18%). *BEST1*-mediated glutamate release has been shown to modulate neuronal excitability, synaptic transmission, and synaptic plasticity [102]. Altered epigenetic regulation of genes involved in calcium influx suggests altered regulation of intracellular calcium concentrations in the hippocampus of pigs suffering from reduced cognition. This hypothesis is further supported by the observed hypomethylation of *INPP5A* (−14.72%), a Inositol-1,4,5-trisphosphate 5-phosphatase involved in regulating calcium concentrations in the hippocampus [103]. Finally, differential methylation of two calcium binding proteins, *SDF4* (−15.9%) and *EFCAB6* (10.94%), was observed. *SDF4* has been previously implicated in neural development [104], as has *SYT9*, which functions as a synaptic calcium sensor for neurotransmitter release [105] and was hypermethylated in the reduced cognition group (25.05%).

## 4. Discussion

The hippocampus plays a critical role in spatial learning and memory and undergoes significant growth and maturation during the neonatal period. Environmental insults during this developmental timeframe can result in altered epigenetic patterns that have lasting effects on brain structure and function. This study assessed hippocampal DNA methylation and gene transcription in pigs suffering from reduced cognitive development in response to two independent early life environmental insults, neonatal viral infection and iron deficiency [39,40,47,48]. By profiling pigs subjected to two different environmental insults during the same developmental period, this study identified conserved molecular mechanisms underlying hippocampal-based spatial learning and memory deficits. Altered DNA methylation and gene transcription associated with neurodevelopment and function, angiogenesis, BBB permeability, and immune activation were detected in this study, providing evidence for the role of epigenetic regulation in early life environmentally-induced reductions in cognitive development.

A number of GO terms enriched for DEGs in this study were associated with activation of immune responses. This result was expected in the case of pigs exposed to early life viral infection. Although iron plays an essential role in immunosurveillance due to its ability to promote growth and differentiation of immune cells [106], pigs exposed to early life iron deficiency were not expected to display significant immune activation. The results of the PCA analysis, including high log2 fold change differences in the expression of genes associated with defense responses between the PRRSv infected and iron deficient pigs, suggests the observed alterations related to immune responses are predominately attributable to changes in the PRRSv infected pigs. However, further investigation into the differences between these two groups is required to confirm this hypothesis.

In an effort to eliminate sex related variability in methylation and expression within groups, only female individuals were utilized in this study. Controlling for sex related variability is especially important in a study such as this given the assessment of pigs exposed to two distinct early life environmental insults, as well as the relatively small sample size of each group. However, because of the exclusion of male individuals, further studies incorporating male subjects are required to determine if the reported mechanisms are sex specific, especially given the known effects sex hormones have on hippocampal function and development.

Consistent with our previous publication [48], altered methylation and expression of genes associated with angiogenesis and BBB permeability was observed in this study. One of these genes, *VWF*, was both differentially methylated and expressed. *VWF* is an endothelial marker expressed at elevated levels in the hippocampus of alcoholics [107]. In addition, higher numbers of endothelial cell clusters expressing *VWF* have been reported in the hippocampus of fetal mice exposed to maternal choline deficiency [108], which results in reduced spatial memory [109,110]. Previous studies have also shown *VWF* knock-out mice maintain tighter blood brain barriers than WT, and that this tighter barrier is actually detrimental to individuals under stressful or diseased states [61]. While the increased *VWF* expression may not be the main cause of the observed reduced cognition phenotype, it does suggest increased blood brain barrier permeability in the reduced cognition groups in response to early life environmental insults.

*VWF* and *LRRC32* represent the only genes that were both differentially methylated and expressed in this study. The low overlap between differentially methylated and expressed genes is likely due to a number of factors, including the use of RRBS as opposed to whole genome bisulfite sequencing (WGBS). While more DMRs associated with DEGs may have been identified using WGBS, which allows for a more thorough profiling of DNA methylation levels at CpG sites across the genome, the increased costs associated with WGBS limited its use in this study. Another factor contributing to the limited overlap between DEGs and DMRs is that DNA methylation is one of many epigenetic regulatory mechanisms that work together to regulate gene expression levels. Therefore, incorporation of chromatin immunoprecipitation (ChIP)-seq or other epigenetic profiling techniques may provide further insights into the epigenetic mechanisms resulting in the differential expression observed in this study. Finally, the relatively low sample size likely limited the power of the study, as it both limited the power of the study and the ability to compare individual groups using the Boruta method. Therefore, further studies utilizing additional individuals are required to confirm the results presented here, as well as further investigate the link between epigenetic alterations and hippocampal based spatial learning and memory.

Although no GO terms related to neurodevelopment and function were enriched for DEGs, differential methylation and expression of a number of genes associated with neurodevelopmental disorders or learning disabilities was observed. The altered expression of genes involved in speech and language development is consistent with recent publications suggesting a role of the hippocampus in language processing [111]. *SETBP1* is also associated with speech impairments, and was differentially methylated in this study. Given the observed reductions in hippocampal-based spatial learning and memory, it is possible that these early life environmental insults could also contribute to delayed language development observed in humans. Upregulation of *PMCH* and *DRD1*, which are involved in regulating behavior responses including social cognition, reward processing, and decision making [69,70,71] was also observed in the reduced cognition group, in addition to altered methylation and expression of a number of genes implicated in neurological and memory disorders. The altered methylation and expression of these genes provides further evidence for their role in neurodevelopment and function as well as a better understanding of the mechanisms underlying reduced cognitive development in response to early life environmental insults.

Finally, reduced expression of *NGF*, which is produced by the hippocampus and is essential for hippocampal plasticity and learning [84], was observed in the reduced cognition group. Reduced *NGF* expression has been previously shown to result in cholinergic and sympathetic neuronal atrophy [85,86,87,88]. Consistent with this result, altered methylation of five genes involved in cholinergic synaptic signaling (*GNG13, PIK3R5, KCNJ6*, *KCNJ5*, and *AKT2*) were differentially methylated in the reduced cognition group. The combination of cholinergic neuronal atrophy, differential methylation of key cholinergic signaling genes, and altered epigenetic regulation of genes involved in glutamate and calcium transport suggests altered cholinergic synaptic signaling represents the main mechanism underlying the reduced hippocampal-based spatial learning and memory observed in response to early life environmental insults, although further work is required to confirm these results. Together, the results of this study suggest that exposure to differential early-life environmental insults results in alteration of conserved molecular mechanisms leading to reduced hippocampal-based spatial learning and memory.

## Supplementary Materials

The following are available online at https://www.mdpi.com/2073-4425/11/2/162/s1, Figure S1: Distribution of methylation regions.
